# Combination therapy using human papillomavirus L1/E6/E7 genes and archaeosome: a nanovaccine confer immuneadjuvanting effects to fight cervical cancer

**DOI:** 10.1038/s41598-020-62448-3

**Published:** 2020-04-01

**Authors:** Hesam Karimi, Hoorieh Soleimanjahi, Asghar Abdoli, Razieh Sadat Banijamali

**Affiliations:** 1 Department of Virology, Faculty of Medical Sciences, Tarbiat Modares University, Tehran, Iran; 2 Department of Hepatitis and AIDS, Pasteur Institute of Iran, Tehran, Iran

**Keywords:** Drug development, Drug development, Tumour virus infections, Tumour virus infections, Adjuvants

## Abstract

Cancer is a leading cause of death worldwide. Cervical cancer caused by human papillomavirus (HPV) is a major health problem in women. DNA vaccines are a perfect approach to immunization, but their potency in clinical trials has been insufficient for generating effective immunity, which may be related to the degradation of the DNA via nucleases, poor delivery to antigen-presenting cells (APCs), and insufficient uptake of DNA plasmids by cells upon injection. Archaeosome is a nano-delivery systems based on liposomes with their immunological role have been developed for gene delivery. In this study, human papillomavirus type 16 genes, containing truncated L1, E6, and E7, were simultaneously used in combination therapy with archaeosome and assessed *in vivo*. Findings supported that archaeosomes promotes immune responses to DNA vaccines and a long-term CTL response was generated with a low antigen dose. Combination therapy with archaeosome/L1/E6/E7 vaccines exhibited a strong cytolytic activity against tumor cells and induced prophylactic and therapeutic effect against the development of tumor in the animal model.

## Introduction

More than four decades have elapsed since the preliminary hypothesis concerning the possible role of human papillomavirus (HPV) as etiological agent of human cancers was suggested. Nowadays, HPV-16 and -18 accounts for approximately 76.7% of cervical cancer worldwide^1^. Due to issues associated with HPV biology and its pathogenesis, progress in developing effective therapies against infection of these viruses has been slow^2^. Numerous therapies are accessible for the management of HPV-related diseases, including surgery, cryotherapy and trichloroacetic acid. Although these strategies are effective, but cannot necessarily eradicate HPV infection as they are applicable for removing only the visible lesions^3^. Since the diagnosis of HPV happens at the late stages of this disease, as little as 7.7% of women demonstrate this infection without any clinical lesion^4^.

Due to the high frequency of infection following the current management approaches, it is crucial to explore substitute methods for an effective inhibition of the cervix and other HPV-related premalignant and malignant lesions^5^. Future strategies to combat HPV may rely on therapeutic vaccines, oncolytic virotherapy, immunotherapies as well as antivirals, natural derivatives/herbs, and therapies based on RNA interference, which are being thoroughly utilized and can interfere with several stages of the viral life cycle. Several of these therapeutic agents are in advanced stages of clinical assessment^5^. HPV vaccine is considered as an encouraging approach to hinder HPV related infection. Although the immunologic effect is not always sufficient to prevent of cancer progression, vaccines are commonly well tolerated and offer valuable anticancer effects in some situations. Several types of vaccine may be used to treat cancers, such as adjuvant and DNA vaccines^6^.

Currently, there are three HPV prophylactic vaccines vaccines based on L1 virus-like particles (VLPs) (Cervarix® and Gardasil®) which are authorized for the prevention of cervical cancer with some limitations. First, HPV protection is type-restricted and more than 200 different types of HPV expressing distinct L1 major capsid proteins. Second, the existing HPV vaccines do not confer therapeutic effect. The third limitation is the relatively high cost of production that prevents their widely use in low- and middle-income countries where nearby 90% of deaths from cervical cancer happens.

Hence, there is a vital necessity for an efficient therapeutic vaccine through which HPV related neoplasia can be eliminated without any surgical treatment^7^. The strategy of somatic gene therapy, the use of DNA as a therapeutic candidate, is an excellent approach to open new horizon for medical applications. Many clinical trials have been conducted in this field. In this method the internal production of antigen let the antigen to be presented to the immune system by means of MHC I antigen-processing machinery while still allowing the MHC-II molecules to act. The production of intracellular antigens via exogenous gene more closely mimics live infections, so they can develop the coordinated triggering of both potent cell-mediated and antibody protective immune responses. Furthermore, DNA vaccines administration in humans has already been demonstrated to be safe and well-tolerated^8^.

The expression of late HPV capsid proteins (L1) occur merely in differentiated epithelial cells, whereas HPV E6 and E7 are expressed throughout the layers of epithelium and act as viral oncoproteins that not only cause carcinogenesis, but also are mainly expressed in HPV-associated pre-malignant and malignant cells. In this view, the simultaneous gene therapy via HPV viral oncoproteins, E6, E7 and L1 major capsid protein, can be a well-combined model for immunotherapeutic approaches for stimulation of immune responses to prevent current HPV-16 related malignancies^9^.

Despite naked DNA plasmid raise measurable levels of antigen-specific immunity and being successful in some preclinical studies; their efficacy in clinical trials has been unsatisfactory for generating effective immunity. This low immunogenicity may be due to the DNA degradation via nucleases, inadequate uptake of DNA plasmids by cells, and poor delivery to antigen-presenting cells (APCs) following injection. Therefore, both viral and nonviral vectors have been utilized to develop delivery systems that offer protection to pDNA for improving the efficacy of DNA vaccination strategies. Effective nonviral delivery systems, however, remains a major problem to clinical gene therapy^10^.

Nano-delivery systems based on liposomes have been the first systems being elucidated to provide adjuvant function. Their immunological character has also been advanced for gene delivery. This role supports that they could boost immune responses to DNA vaccines through enhancing their uptake via APCs in the lymphoid tissues.

Liposomes composed of synthetic esters have been explored as drug or antigen carriers, and a nano liposome-based vaccine against hepatitis A being licensed for use in humans^11^. The main limitation to the application of conventional liposomes is their instability, relatively short half-life and uneconomical production especially in large scales. Conventional liposomes can be quickly removed from the circulation by liver Kupffer cells and spleen macrophages, vesicle disruption at low pH in the gastrointestinal areas and lipid hydrolysis because of lipases^12^.

Archaeosomes belong to a biological liposomes family which are composed of total polar lipids extracted from archaea, such as methanogens, halophiles and thermoacidophiles^13^. Archaeal lipids are composed of typically fully saturated and branched phytanyl chains in many species which are being linked through ether linkage to the glycerol backbone at the positions of the sn-2, 3. The unique structures of archaeal lipids confer considerable stability on archaeosomes. These remarkable molecular and physicochemical characteristics of archaeal lipids confers some superiority over the custom ester phospholipid liposomes which led to the adaptation of their membrane lipid compositions to stringent environments, suitable for the highly stable archaeosomes preparation^14^. Archaeosomes are demonstrated extremely effectual as a self-adjuvanting vaccine strongly eliciting antigen-specific, both cell-mediated immune and humoral responses, to encapsulated antigens. Some superior characters of Archaeosomes compared with conventional liposomes and other adjuvants are the stimulation and recruitment of dendritic cells; antigen orientation to process via MHC class I which leads to the potent activation of CD8 + cytotoxic T lymphocyte responses that is pivotal to combat against intracellular pathogens and cancer; and archaeal lipid cores stability which make a profound immune memory easier^15,16^.

Additional significant advantages of archaeosomes include a low number of boosts required to reach a high circulating antibody titer. Archaeosomes are safe without any noticeable toxicity. They are more resistant to chemical hydrolysis, oxidation, bile salts, acidic or alkaline pH, and high temperatures with various vaccine and drug delivery applications^17^. Therefore, liposomes formed by archaebacterial membranes lipids represent a promising alternative to the classical liposomes and virosoms, consequently found extensive applications in vaccine and drug delivery^18–20^.

With these in mind, the current study aimed to assessment the ability of the cellular antigenic regions of the HPV 16 genes, containing truncated L1, E6, and E7, used simultaneously in combination therapy with archaeosomes as an adjuvant and nanoparticle carriers. In this study, archaeosome was prepared and characterized, then formulated with DNA vaccine (L1/E6/E7 recombinant genes). In the next step, the cervical cancer tumor model was provided in C57BL/6 mice and the ability of the vaccine in stimulating different facets of immune responses evaluated; also, the DNA fragmentation in tumor cells following administration of DNA vaccine/archaeosomes formulation in animal models was assessed.

## Results

### *In vitro* detection of pIRES-L1/E6/E7-mediated GFP gene expression

The recombinant DNA vaccine candidate (pIRES- L1/E6/E7) utilized in this study was generated in our previous study and validity of synthesized sequences was confirmed using bioinformatics software and western blot. The list of the amino acid sequences of the peptides can be found as Supplementary Table S1. The final DNA sequences was designed and optimized using bioinformatics software (see Supplementary Table S2). Synthesized gene sequences encoding cellular antigenic epitopes of the L1, E6 and E7 create 397 bp.

In current study, to confirm the expression of the recombinant plasmid carrying the L1/E6/E7 gene, we examined the GFP protein expression using a fluorescence microscope.

**Figure 1:**
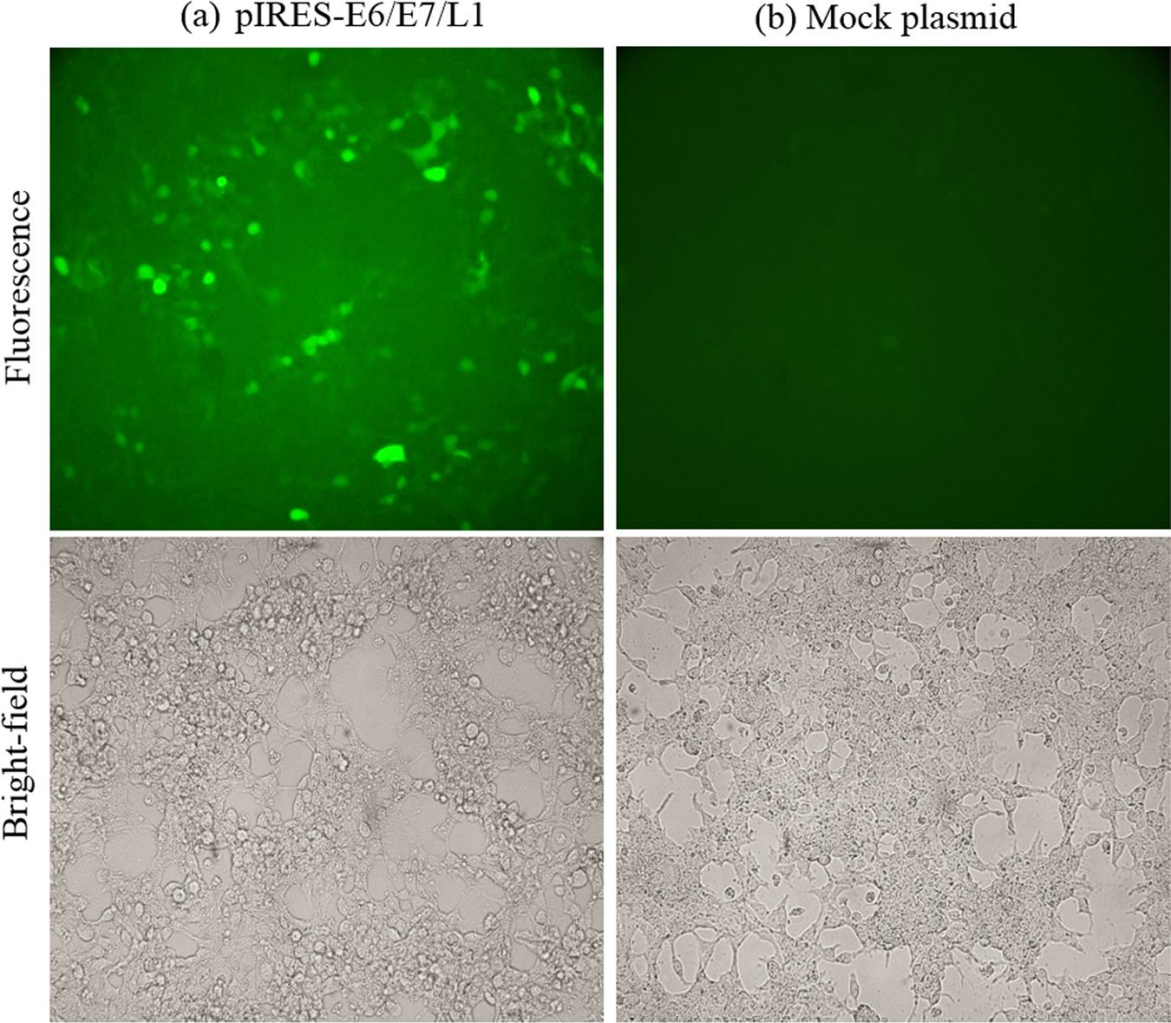
Fluorescence microscopic observation of pIRES-L1/E6/E7 expression. HEK293T cells were transfected with pIRES-L1/E6/E7 and Mock plasmids. (**a**) GFP expression was seen 24 hours after HEK293T cells were transfected with pIRES-L1/E6/E7, (**b**) but not in Mock plasmid.

The recombinant DNA vaccine candidate (L1/E6/E7) was subcloned into pIRES2-EGFP vector. This vector contains the internal ribosome entry site (IRES; 1, 2) of the encephalomyocarditis virus (ECMV) between the Multiple Cloning Site (MCS) and the enhanced green fluorescent protein (EGFP) coding region. This permits both the gene of interest (cloned into the MCS) and the EGFP gene to be translated from a single bicistronic mRNA. Therefore, the pIRES- L1/E6/E7 plasmid was utilized for the HEK293 cell transfection assay. After 24 h of culture, the GFP protein expression was visualized using a fluorescence microscope (Fig. 1). The negative control did not demonstrate any detectable fluorescence.

The construct expression in HEK293 cell line was verified via western blot. The applied primary antibodies included mouse monoclonal Anti-HPV16 L1 antibody [CamVir 1] (ab69). Secondary antibodies were goat anti- Mouse IgG H&L (HRP) (Abcam). Binding signals were visualized with TMB substrate (Sigma-Aldrich, T0565). The result of western blot provided as a supplementary file (see Supplementary Fig. S1).

### Vesicle visualization by TEM

**Figure 2:**
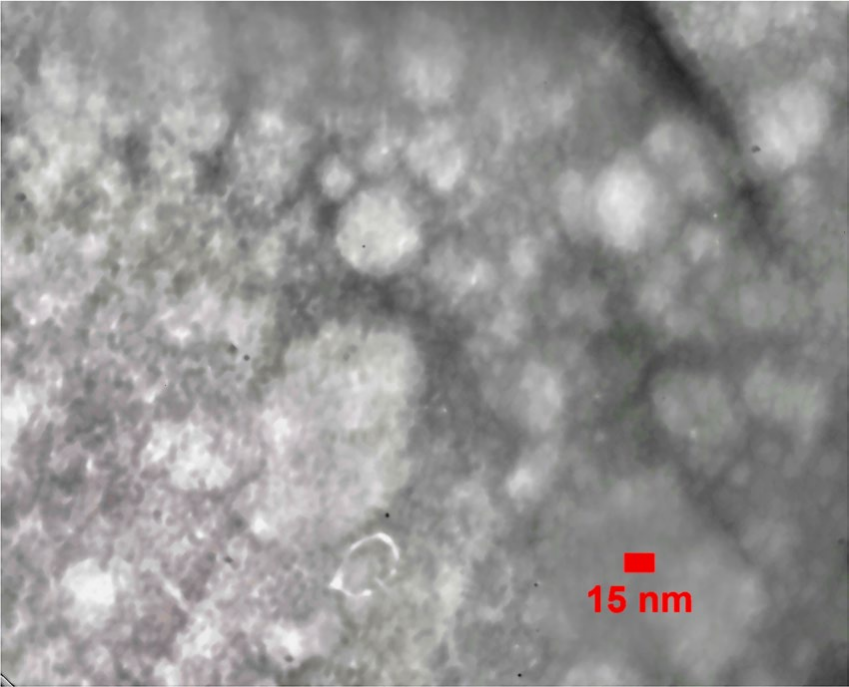
TEM micrographs of archaeosomes are shown. Purified archaeosomes were negatively stained with 2% uranyl acetate and observed under TEM (ZIESS, EM900, PHILPS, Germany). Archaeosome were approximately between 50 and 100 nm in diameter.

According to Fig. 2, the TEM micrographs of uncomplexed archaeosomes present a spherical shape as well as homogeneous size distribution. As indicated in Fig. 2, the size of vesicles ranged between 50 and 100 nm.

### Physicochemical characterization of archaeosome

Mean size and zeta potential of vesicles are given in Table 1. The zeta potential of uncomplexed archaeosomes was nearly zero (−6.84 ± 0.8 mV), whereas the addition of pDNA to the archaeosome suspension caused a change in the zeta potential and particle size of archaeosomes.

**Table 1:** Physicochemical characterization of archaeosomes (mean ± SD, n = 3).

Archaeosome formulation	Z-potential (mV)	Mean size
Archaeosomes alone (antigen-free archaeosomes)	−6.84 ± 0.8	98.1 ± 1.3
pDNA-surface localized archaeosome	−29 ± 0.7	127 ± 2.1
pDNA-encapsulated archaeosome	−30.8 ± 0.7	429 ± 5

The pDNA-surface localized archaeosome and pDNA-encapsulated archaeosome had a zeta potential of −29 ± 0.7 and −30.8 ± 0.7 mV, respectively. Apparently, the helper molecules, CaCl_2_, improved electrostatic interaction between pDNA and archaeosome. Moreover, the addition of pDNA to archaeosome caused a significant change in the particle size. In uncomplexed vesicles, average particle size was 98.1 ± 1.3 nm, whereas in the case of formulations containing pDNA-surface localized archaeosome and pDNA-encapsulated archaeosomes the average particle size was 127 ± 2.1 nm and 429 ± 5 nm, respectively.

### L1/E6/E7- archaeosome immunization induces a potent specific CD8** +** T-cell response

**Figure 3:**
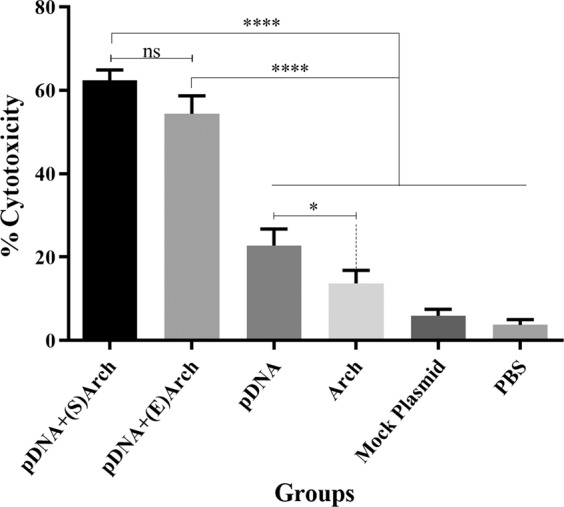
Cytotoxic activity of splenocytes against TC1 tumor cells. Mice were vaccinated three times with the each vaccine group or controls at one-week intervals, following inoculation with TC1 tumor cells. The splenocytes were co-cultured with TC1 cells for 6 to 8 h at an effector to target ratio (E:T) of 25:1. The CTL activity of the splenocytes was significantly increased in the mice immunized with pDNA-encapsulated archaeosomes and pDNA-surface localizated archaeosome group compared with that of the pDNA alone (pIRES-L1/E6/E7) and control groups (P < 0.0001). Data on the cytotoxicity induced by different treatments are presented as mean ± SD calculated from three experiments. (Abbreviations: pDNA + (S)Arch: pDNA-surface localized archaeosome; pDNA + (E)Arch: pDNA-encapsulated archaeosomes; pDNA: pDNA alone (L1/E6/E7 recombinant gene); Arch: archaeosomes alone (antigen-free archaeosomes); Mock Plasmid: pIRES.

To determine whether immunization with various L1/E6/E7- archaeosome formulations induced cytolytic activity against TC-1 cells, one week after the last immunization, a single-cell suspension of splenocytes from each mouse was prepared and assayed for cytolytic activity. Generation of effector CD8 T cells (CTLs) was assayed in the present study by the LDH release assay in triplicate in 96-well plates by culturing the effector cells with TC-1 target cells at the effector/target (E/T) ratio of 25:1. The data presented in Fig. 3 indicate that effectors cells derived from mice immunized with pDNA-encapsulated archaeosomes and pDNA-surface localized archaeosome were significantly more cytotoxic for TC-1 targets than those obtained from other vaccine and control groups (p < 0.0001). Data on the cytotoxicity induced by different treatments are presented as the mean ± SD calculated from three experiments.

### L1/E6/E7-archaeosome induce Th1 and Th2 cytokine production by Ag-stimulated spleen cells

To evaluate the frequency of T cell immune responses induced by various formulation of vaccines and investigation of the potential mechanisms responsible for the therapeutic efficacy of the vaccine candidate on TC-1 tumor, the proportion of different cytokines between experimental groups was compared.

Tumor-bearing C57BL/6 mice were immunized three times at one-week intervals using each DNA vaccine alone or in combination with the archaeosome (pDNA-encapsulated archaeosomes and pDNA-surface localized archaeosome).

**Figure 4:**
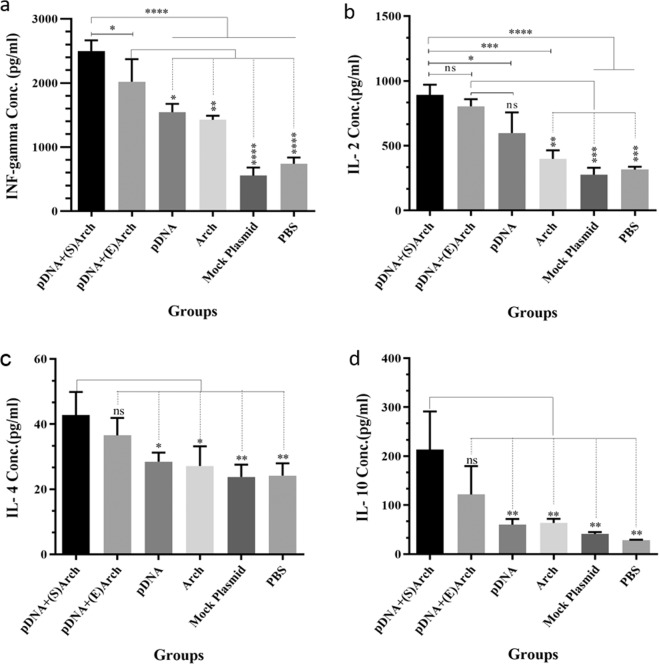
The level of cytokine production by splenocytes of immunized mice. Splenocytes obtained from immunized mice were cultured in triplicate for each mouse and stimulated with appropriate mitogen. After 72 h supernatants were collected and analyzed by sandwich ELISA method to measure (**a**) IFN-γ, (**b**) IL-2, (**c**) IL-4 and (**d**) IL-10 levels separately. The data are shown as mean ± S.D responses of each group. The levels of statistical significance for differences between experimental groups were determined using ANOVA followed by Turkey’s post-test. The ns indicates not significant difference between groups. The asterisks indicate the groups, which were significantly different. *(P < 0.05), **(p < 0.01), ***(P < 0.001) and ****(P < 0.0001). (Abbreviations: pDNA + (S)Arch: pDNA-surface localized archaeosome; pDNA + (E)Arch: pDNA-encapsulated archaeosomes; pDNA: pDNA alone (L1/E6/E7 recombinant gene); Arch: archaeosomes alone (antigen-free archaeosomes); Mock Plasmid: pIRES.

Seven days after the last immunization, freshly isolated splenocytes of immunized mice were cultured in the presence of different mitogens *ex vivo*, and various cytokine profiles such as IFN-γ, IL-2, IL-10, and IL-4 secreted via T cells were assessed by ELISA kits. As shown in Fig. 4(a,b), splenocytes taken from immunized mice receiving pDNA-surface localized archaeosome and pDNA-encapsulated archaeosome vaccine groups produced higher levels of Th1-cytokine, IFN-γ and IL-2, in comparison to other groups, including the pDNA alone, antigen-free archaeosomes, PBS and mock plasmid (pIRES alone) groups. In two main vaccine groups, the concentration of IFN-γ was significantly higher in the splenocytes culture medium of mice immunized by pDNA-surface localized archaeosome in comparison to the group immunized by pDNA-encapsulated archaeosomes.

The pDNA alone (L1/E6/E7 recombinant gene) induced IFN-γ production compared with control groups, and the rate of this stimulation was significantly increased after being formulated with archaeosome, representing the adjuvant and protective properties of archaeosome nanoparticles. Furthermore, the number of IFN- γ- and IL-2-secreting splenocytes in mice immunized with antigen-free archaeosomes was statistically significant and approximately two-fold higher than in the rest of the mock plasmid and PBS control groups, demonstrating the intrinsic adjuvant property of the archaeosome nanoparticles.

Data in Fig. 4(c,d) indicate that the CD4 + T-cells from animals immunized with pDNA-surface localized archaeosome and pDNA-encapsulated archaeosomes groups, exhibited remarkable levels of proliferation and secreted Th2 cytokines, IL-4 and IL-10, into the culture supernatant. The frequency of IL-4 and IL-10-secreting cells in the pDNA-surface localized archaeosome groups was significantly higher than other groups, while the frequency of these splenocytes in pDNA-encapsulated archaeosome and pDNA-alone immunized groups showed no significant difference compared with control groups. The asterisks indicate the groups, which were significantly different. *(P < 0.05), **(p < 0.01), ***(P < 0.001) and ****(P < 0.0001).

### Effect of treatments on apoptosis in a cervical cancer model

To assess the possibility that the administration of vaccines candidate would affect apoptosis in the cervical cancer model, the tumor cell was analyzed following vaccination with L1/E6/E7 gene with or without the formulation of archaeosome. For this purpose, DNA fragmentation in tumor cells was examined by TUNEL assay in pDNA-surface localized archaeosomes, pDNA-encapsulated archaeosomes, L1/E6/E7 recombinant gene alone, and the results were compared with DNA fragmentation from PBS control groups. Nuclear staining was observed using a DAPI filter (magnification for g through n: 40X). All vaccine treatments displayed an elevated number of apoptotic cells by TUNEL assay when compared to control groups.

The majority of these apoptotic cells were found in the tumor following pDNA-surface localized archaeosome and pDNA-encapsulated archaeosome treatment groups. The number of apoptotic cells in tumors significantly decreased following L1/E6/E7 recombinant gene vaccine treatment alone compared to that following the main treatments.

**Figure 5:**
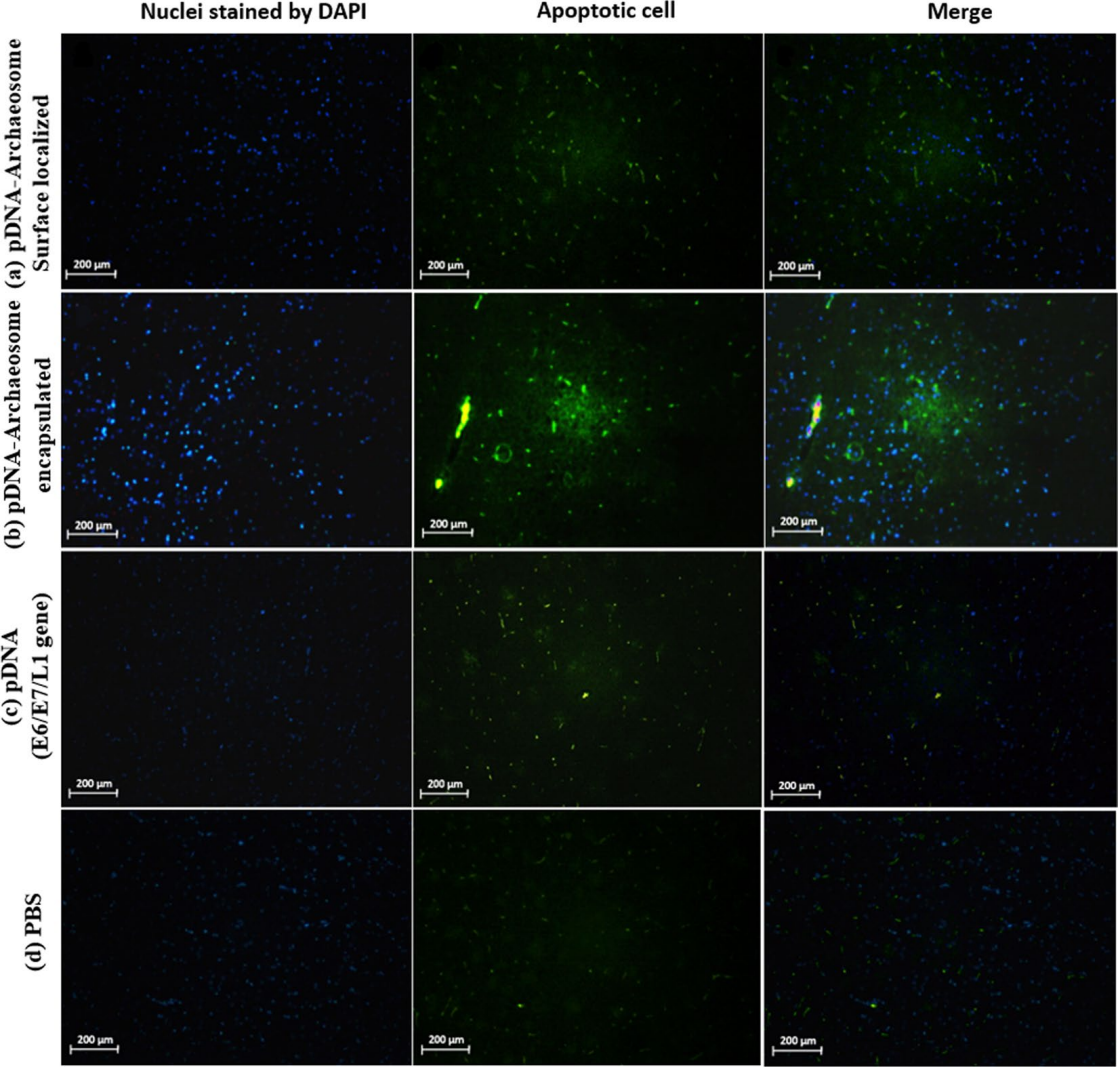
Tumor cell apoptosis in vaccinated mice. For the analysis of the apoptosis marker *in vivo*, TC-1 tumor-bearing animals were vaccinated with each vaccine groups or PBS. Tumor apoptosis was detected using TUNEL and analysed by fluorescence microscopy in (**a**) pDNA-surface localized archaeosomes group, (**b**) pDNA-encapsulated archaeosomes group, (**c**) L1/E6/E7 recombinant gene alone group and (**d**) PBS control group. Total nuclei are visualized in blue (DAPI) and TUNEL-positive nuclei are stained in green (FITC). The images are representative of three independent experiments. TUNEL analysis indicated that the combined L1/E6/E7 + archaeosomes treatment was associated with more apoptosis (40–60%) than the L1/E6/E7 alone (15–20%). Controls (no treatment) showed a low background level of apoptosis (0–1.5%). All images are shown at × 40 magnification.

Therefore, according to the results, treatment with the combination of L1/E6/E7 genes alongside archaeosome nanoparticle was associated with the greatest extent of apoptosis, ranging from 40–60%. Treatment with L1/E6/E7 recombinant gene led to less apoptosis, ranging from 15–20% for pDNA alone. Finally, treatment controls resulted in background apoptosis levels ranging from 0 to 1.5%. These results suggest a synergistic effect between L1/E6/E7 gene and archaeosome vaccines individually (Fig. 5).

### Therapeutic immunization with L1/E6/E7 - archaeosome vaccine induces a potent antitumoral activity

In order to study the potency of the archaeosome nano-vaccine, mice with pre-established TC-1 tumor models were treated with either vaccine, and tumor volume were monitored in different groups for 28 days following the challenge.

**Figure 6:**
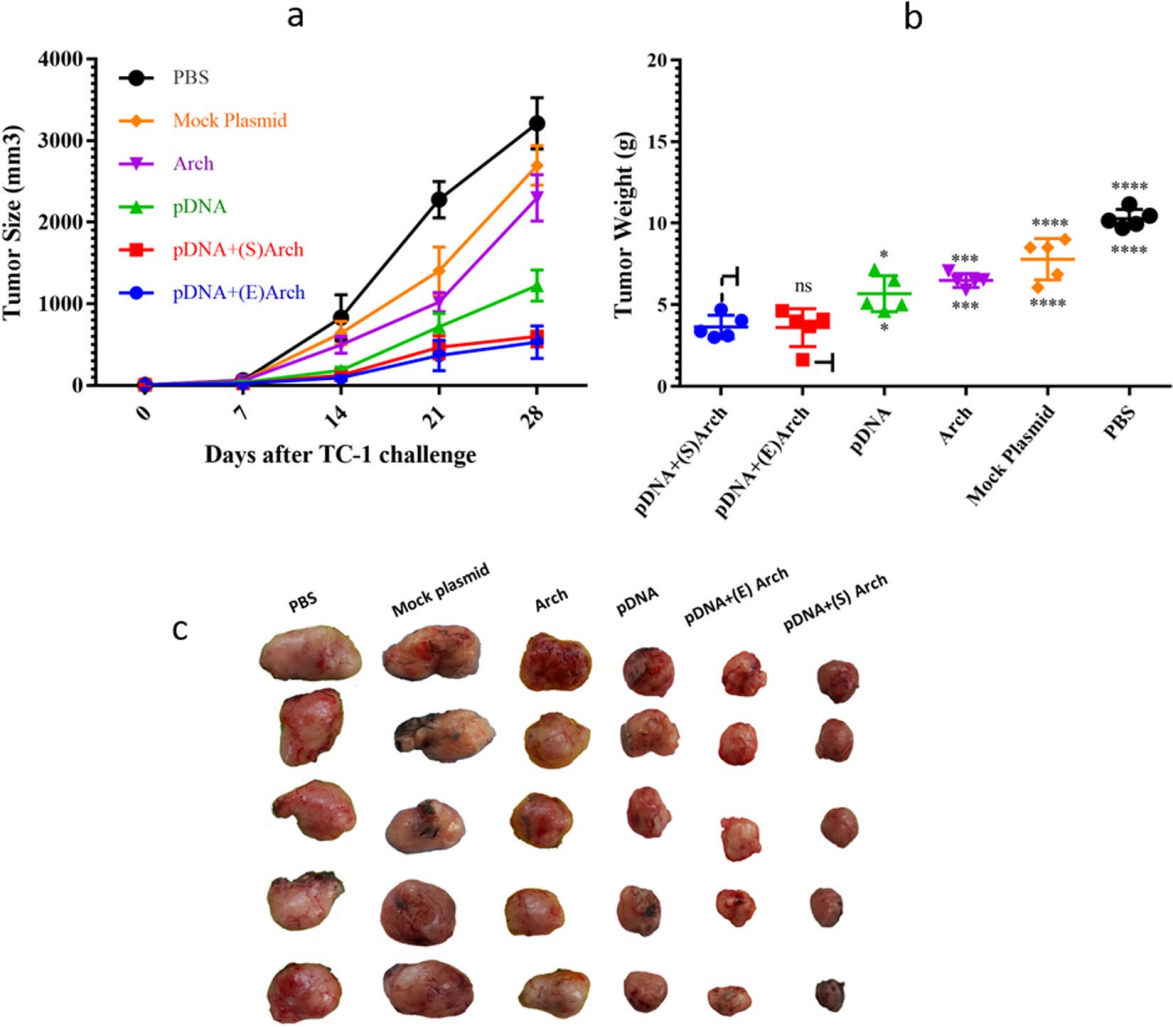
Therapeutic immunization with L1/E6/E7 - archaeosome vaccine inhibited tumor growth *in vivo*. Mouses were vaccinated three times with the different formulation of L1/E6/E7 - archaeosome vaccines and controls at weekly intervals following the challenge with TC-1 tumor cells. The tumor volume was monitored three times a week for 28 days. (**a**) Growth of TC1 tumors in mice treated with vaccines. The volume of the tumors in the groups which tumors had been treated with L1/E6/E7 recombinant gene plus archaeosome was significantly smaller compared with that of the PBS and other control groups (P < 0.0001). (**b**) The mean tumor weight of the L1/E6/E7 - archaeosome vaccine groups were significantly lower compared with that of the control groups on day 28. The asterisks indicate the groups, which were significantly different. *(P < 0.05), ***(P < 0.001) and ****(P < 0.0001). (**c**) Mice immunized with archaeosome/pDNA nanoparticles demonstrated a significant difference in tumor growth in comparison to other groups. (Abbreviations: pDNA + (S)Arch: pDNA-surface localized archaeosome; pDNA + (E)Arch: pDNA-encapsulated archaeosomes; pDNA: pDNA alone (L1/E6/E7 recombinant gene); Arch: archaeosomes alone (antigen-free archaeosomes); Mock Plasmid: pIRES.

Based on Fig. 6(a), the average tumor volume was in the range of 25–65 mm^3^ on Day 7 (initiation of treatment). Seven days after the end of administration (Day 28), the average tumor volume was 601.9 ± 125.67 mm^3^ and 530 ± 198.09 mm^3^ in the pDNA-surface localized archaeosome and pDNA-encapsulated archaeosome group, respectively; 1225.4 ± 190.68 mm^3^ in the L1/E6/E7 recombinant gene alone group; 2297.5 ± 284.15 mm^3^ in the antigen-free archaeosomes; and 2696.28 ± 240.86 and 3212 ± 314.62 mm^3^ in the mock plasmid and PBS control group, respectively.

Although tumor growth in the L1/E6/E7 recombinant gene alone group was much slower than the mock plasmid and PBS control groups after treatments (P < 0.0001) (Fig. 6a); and the average volume on Day 28 was 30% of that with PBS control group treatment. Groups in which tumors had been treated with L1/E6/E7 recombinant gene plus archaeosome tended to decrease in size and significantly delayed the growth of tumors in mice, so that the average volume on Day 28 was 15% of that with PBS control group. Therefore, *in vivo* anticancer efficacy studies demonstrated that the combination treatment with archaeosome-pDNA formulation greatly hindered the tumor progression compared to each treatment alone.

In addition, a statistically significant difference was observed in tumor weight between pDNA-surface localized archaeosome and pDNA-encapsulated archaeosomes groups-treated mice and other groups. Tumors treated with PBS and mock plasmid control group reached 10.28 ± 0.5 g and 7.79 ± 1.2 g, respectively. However, tumor weight was reduced to 3.64 ± 0.7 g and 3.6 ± 1.1 g (Fig. 6b) in the pDNA-surface localized archaeosome and pDNA-encapsulated archaeosomes group, respectively. The asterisks indicate the groups, which were significantly different: *(P < 0.05), ***(P < 0.001) and ****(P < 0.0001). Mice were euthanized when tumor diameter exceeded>5% of body weight. As indicated in Fig. 6(c), mice immunized with archaeosome/pDNA nanoparticles demonstrated a significant difference in tumor growth in comparison to other groups.

### Therapeutic immunization via L1/E6/E7- archaeosome vaccine promotes long-term survival

In this study there were six animal groups, and each group consisting of eight pathogen-free female C57BL/6 mice. Five Mice of each group were euthanized one week after the 3rd immunization (i.e., Day 28) and spleens and tumor were collected for the evaluation of different experiment. Three mice remained in each group were closely monitored up to 42 days and the cumulative survival rate was calculated.

**Figure 7:**
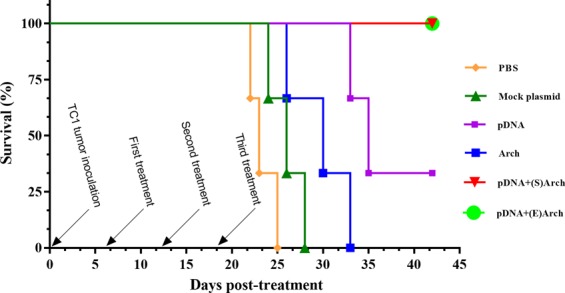
The Kaplan-Meier survival curves demonstrate long-term protection from tumor outgrowth by Therapeutic immunization with vaccines. The mice were monitored for 42 days and the cumulative survival rate was calculated. Both pDNA-surface localized archaeosome and pDNA-encapsulated archaeosomes were able to suppress TC1 tumor growth and the survival time of mice in those groups was prolonged, compared with that of the other groups. (P < 0.0001). (Abbreviations: pDNA + (S)Arch: pDNA-surface localized archaeosome; pDNA + (E)Arch: pDNA-encapsulated archaeosomes; pDNA: pDNA alone (L1/E6/E7 recombinant gene); Arch: archaeosomes alone (antigen-free archaeosomes); Mock Plasmid: pIRES).

Figure 7 depicts the results obtained when the mice receiving either pDNA-surface localized archaeosome and pDNA-encapsulated archaeosomes and L1/E6/E7 recombinant gene survived longer than those in the control group (P < 0.0001). Nevertheless, a considerable level of protection and survival time was obtained with L1/E6/E7 in the formulation with archaeosome, suggesting that a combination of archaeosome/L1/E6/E7 may provide slightly superior protection than pDNA alone. At a time when no mice survived in the PBS and mock plasmid groups (i.e., Day 42), 100% of mice in the pDNA-surface localized archaeosome and pDNA-encapsulated archaeosomes groups and 33.33% of mice in the L1/E6/E7 recombinant gene group were still alive (Fig. 7).

## Discussion

Despite advances in the treatment of cancer, nearly 530,000 new instances of disease and 275,000 deaths from cancer occur annually worldwide. Viruses play a significant role in many human cancers^21^. It is now estimated that 20–25% of human cancers worldwide have a known viral etiology^22^. Human papillomavirus plays essential roles in the initiation as well as progression of cervical cancer, and continued expression of their viral transforming activities is required for the maintenance of the transformed phenotype^4^. Investigating of the association between the high-risk HPV, especially HPV 16 (60–50%), and the development of cervical cancer, initiates a wide range of studies aimed at producing therapeutic or prophylactic vaccines against HPV infection^6,23^. To design a vaccine with a high potential to boost immune responses, considering of several parameters such as the dose, time, and routes of administration are essential. Also an appropriate adjuvant in vaccine formulation to increase the stimulation of the immune system are necessary. Although the infection by high-risk papillomavirus can be prevented with introducing of Gardasil and Cervarix vaccines, but the problem remained still due to the general non- vaccination of communities and the presence of non-responder infected populations^24^. It seems that there is no ideal vaccine with a high efficacy in clinical phases, which can act as a therapeutic and preventive. Therefore, designing these types of vaccine was the aim of the present study.

In viral diseases and cancer, the activation of CTLs to eliminate infected and cancerous cells is necessary. Thus, it is vital to use strategies for activating CTLs^25^. DNA vaccines are a valuable form of antigen-specific immunotherapy which are transcribed in eukaryotic cells using a proprietary viral promoter, and the target gene supply is performed through the MHC-I pathway which activates CTLs and stimulates the host immune system. In addition, the induced immune response is long-term^26^. The cellular immune response in contrast to humoral immunity plays an important role in effectively responding against human papillomavirus. It is believed that the human papillomavirus vaccine should not only prevent the formation of primary infections, but also provide treatment approach. Antibody‐mediated immunity is only able to prevent infection, while cell-mediated immunity against the intracellular proteins of the papillomavirus can perform both functions by activating T helper cells for the proliferation and differentiation of B cells and CD8+ (cytotoxic) T cells and the production of inhibitory cytokines^27^.

In the initial human studies, the protective effect of L1 protein against HPV infection and the therapeutic role of E6 and E7 proteins after exposure to the papillomavirus have been demonstrated^28^. The HPV-E7 oncoprotein is an appropriate candidate for designing anti-tumor vaccines, which in addition to the production of anti-E7 IgG in mice, induces the activity of cytotoxic T cells. Consequently this vaccine has both preventive and therapeutic properties^28^. Liu *et al*. (2002) showed that the immunogenicity of the polynucleotide vaccine increases when using optimized HPV-E7^29^ and, in the case of HPV 16 E7, reported that N-terminus and C-terminus are immunogenic. On the other hand, numerous studies have indicated that HPV16 E6-based vaccines are an effective way to prevent the E6 gene expression in the tumor.

Cheng *et al*. (2006) at Johns Hopkins University concluded that the DNA vaccine containing E6-gene has a strong ability for protein expression in the cell culture models and can more effectively induce CTL responses^30^. Mirshahabi *et al*. (2012) showed that E6 protein of type 16 human papillomavirus can induce cytotoxic T-cell responses in a cell culture model^31^. Since E6 and E7 proteins are continuously expressed at all stages of cervical cancer, they can be employed as an ideal target to produce therapeutic vaccines and develop immunotherapy methods against papillomavirus tumors. Blounet *et al*. (2009) demonstrated that specific T-CD8+ and CD4+ lymphocytes of L1 gene have therapeutic effects in patients with cervical cancer^28^.

In the present study, we used the perfect combined construct contains the immunogenic regions of the papillomavirus L1, E6, and E7 recombinant gene in the formulation of archaeosome nanoparticles as an adjuvant and carrier system to increase and strengthen the immunogenicity of these DNA vaccine. So far, no report has been presented based on the investigation of archaeosome nanoparticles as a carrier and adjuvant in formulation with DNA vaccine for the treatment of cervical cancer in the mice model.

The archaeosome technology is a new self-adjuvant delivery system with the ability of antigen delivery to the MHC class I processing pathway and stimulating the intrinsic immune system to induce a strong and long-term immunity against subunits of antigen^32,33^. Archaebacteria are a group of non-pathogenic organisms not associated with endotoxins or other toxic metabolites, and thus archaeosomes are predicted to be safe^34^. Early studies have demonstrated that archaeosomes can induce a long-term antibody response in their associated antigen only after two initial injections on Days 0 and 21^33^. In the initial stage after immunization by bovine serum albumin-archaeosomes, more than 95% of CD8 + T cells responded to the antigen^15^. The study by Patel *et al*. revealed that the archaeosomes made of lipids extracted from *M. smithii* are an appropriate adjuvant for a multivalent mucosal vaccine. The archaeosome containing encapsulated antigen OVA and egg lysozyme demonstrated a robust and specific antibody response to all antigens^16^. Krishnan *et al*. (2010) reported that archaeosomes made of lipids extracted from *M. smithii* are a strong adjuvants for evoking a CD8 + T cell response. Their results exhibited that system could be used in the formulation of cancer vaccines^13^. Rethore *et al*. (2009) examined the archaeosome made of synthetic tetraether lipids as a new gene delivery system, and the results showed that these archaeosomes could be used for the delivery of genes *in vitro*^35^. Due to their high stability, archaeosomes can be considered as suitable carriers for proteins, peptides, and genes^17^.

Nanoparticle carriers provide adjuvant activity by increasing the delivery of the antigen or activating the inherent immune response. The strength and mechanism of stimulating the immune system induced by nanocarrier vaccines depend on various factors, including chemical composition, particle size, homogeneity, load, nature, the position of the antigen within the carrier, and site of administration (123). DNA-liposome complexes enter the cell through endocytosis. In this method, the cytoplasmic membrane surrounds the liposome like a sheath and creates an intracellular carrier called endosome; liposome is decomposed inside the endosome; and the DNA is released into the cytosol and transferred to the nucleus^36^.

Choosing lipids to make liposomes as carriers for drug or DNA vaccine depends on several factors^37^. The presence of cholesterol is required in stabilizing the dual-layer membrane and increasing the liposomes’ persistence in the *in vivo* environment. In the absence of cholesterol, liposomes are destabilize by HDL and followed by the release of the contents, and the drug is rapidly removed from blood circulation^38^. In the endocytosis process for entering the cells, liposome is swallowed by cells, like the phagocytosis of foreign particles by phagocytes. It has been shown that, by decreasing the size of liposomes, their surface potential is reduced. Also, increasing the particle size of liposomes to more than 1000 nm leads to rapid swallowing by macrophages and, consequently, early removal from blood circulation^39^. Macrophage cells which have different endocytic activities swallow almost all types of liposomes. According to studies by Allen *et al*., elimination of liposomes with a very small size (about 8–4 nm) does not occur, and these particles are only partially removed by glomerular filtration in the kidneys. However, due to their inherent instability, vesicles with a particle size of less than 40–50 nm are not constructed^38^.

In the present study, the nano-archaeosome particle size was optimized and measured by the Zetasizer Nano ZS, which approximately equaled 98.1 ± 1.3 nm before formulation with plasmid and about 429 ± 5 nm and 127 ± 2.1 nm after formulation in the pDNA-encapsulated archaeosomes and pDNA-surface localized archaeosomes, respectively. Studies have shown that immunization with small liposomes induces a Th2 response, whereas large liposomes induce a Th1 response, higher interferon gamma (IFNγ) levels, and immunoglobulin IgG2a/IgG1 ratios^40^. According to the present results, it seems that the size of the nano- archaeosomes is within an appropriate range and, therefore, one of our main goals has been achieved.

In this research, the zeta potential after formulation with plasmid DNA became more negative, reaching −29 ± 0.7 mV from −6.84 ± 0.8 mV at surface formulation and −30.8 ± 0.7 mV at encapsulation form, which can be related to the addition of DNA to archaeosome. In many studies, it has been concluded that the negative charge contributes to a better transfer of DNA into the cell and an increase in transfection^41^.

In order to investigate the ability of the vaccine for the induction of immune response, and the evaluation of the properties of archaeosome nanoparticles *in vivo*; was provided the cervical cancer tumor model in C57BL/6 mice using the subcutaneous injection of TC-1 tumor cells. In many similar studies, the amount of injected DNA vaccine was 100 μg/100 μl per dose. In this study, in order to reduce the vaccine dose and investigate the effect of archaeosome as carriers and adjuvants on increasing the efficacy of low-dose DNA vaccine, it has been shown that fewer amounts of DNA vaccine in formulation with archaeosome significantly reduce the tumor size and stimulate cellular immune responses.

Given the importance of the cytokine response in antiviral and anti-tumor immunity, if the type 1 cytokine response is produced in vaccinated mice, the immune system will be able to eliminate the infected tumor cells. Therefore, in designing therapeutic vaccines, the use of a carrier or adjuvant with the goal of shifting the cytokine balance towards Th1 immune responses and cell-mediated responses should always be considered. Thus, in this research, the cytokine secretion of the splenocytes of the vaccinated mice in test groups was compared with negative control groups. One of the first problems in the use of Alum as an adjuvant has been its inability to induce strong IgG2a and cell-mediated immunity, in particular, Th1 cells. Archaeosome strongly induces both IgG1 and IgG2a^34^. In addition, the antigen-specific activation of both Th1 and Th2 cells was corroborated by splenocytes from archaeosome-antigen immunized mice relative to the antigen Alum group^42^. Archaeosome-antigen presentation occurs through the classical cytosolic pathway and MHC class I molecules, in which a strong adjuvant activity is sufficiently potent to bypass a requirement for CD4 + T cell help. The published results clearly demonstrated the superior adjuvant activity of archaeosome in comparison with conventional liposomes and Alum^42^.

Th1 cells produce cytokines such as IFN-γ and IL-2 which are involved in the differentiation of precursor mouse CD8 + T cells into cytotoxic CD8 + T cells. In this study, IFN-γ secretion in two main vaccine groups, that received the surface localized and encapsulated pDNA/archaeosome vaccines, was significantly higher than that in other groups. Moreover, the DNA vaccine alone (L1/E6/E7 recombinant gene) somewhat induced an IFN-γ-type cytokine response, suggesting that the presence of three truncated E6, E7, and L1 genes together leads to an appropriate immune response. In this research, the rate of immune response increased after the formulation of L1/E6/E7 recombinant gene and archaeosome, representing that the archaeosome provides a potent adjuvant activity and protects the pDNA from degradation and oxidation. This protective phospholipid shield undamaged until the contents of the liposome are delivered to the exact target where the contents will be utilized. In addition, since empty archaeosomes alone with a significant difference in comparison with PBS and mock plasmid groups have caused IFN-γ secretion, one can note the intrinsic adjuvant property of archaeosome, consistent with other studies.

Interleukin-2 plays a determining role in controlling the survival of immature and mature T cells. In this study, groups vaccinated with DNA vaccine-archaeosome formulation have significantly improved IL-2 secretion compared to control groups. Surface-localized and encapsulated pDNA/archaeosome vaccines induced the highest IL-2 production, and these findings can indicate the adjuvant and delivery property of archaeosome, causing appropriate protectiveness and boosting the DNA vaccine efficiency. Since nanoparticles alone induced IL-2, an inherent self-adjuvanting property of archaeosome nanoparticles was well demonstrated in this study.

Interleukin-4 is produced by Th2, NK, and mast cells. In this study, IL-4 cytokine measurement showed that, in groups receiving surface localized L1/E6/E7/archaeosome vaccines, they significantly induced IL-4 secretion in comparison with the control groups. In addition, encapsulated L1/E6/E7 recombinant gene in archaeosome induces IL-4 secretion, but it is not remarkable in comparison to other groups; this can be due to the lack of DNA access in the encapsulated state and the slow release of plasmid from archaeosome.

Based on the results of this study, it was observed that in groups receiving the L1/E6/E7 recombinant gene DNA vaccine in the surface of archaeosome, they significantly induced IL-10 secretion compared to PBS, mock plasmid, L1/E6/E7 recombinant gene, and empty archaeosomes alone groups. As previously stated, IL-10 cytokine is a stimulant for the humoral immune system and stimulates B lymphocytes. In the present study, the surface localized L1/E6/E7/archaeosome vaccines group stimulated the secretion of IL-10 more than the encapsulated L1/E6/E7/archaeosome vaccines group. It can be said that a high-level induction of IL-10 in the form of surface formulation is expected due to the availability and faster release in comparison to the encapsulated state, while the enclosed antigens need a disturbance in vesicles to be available. IL-10 secretion level in the other groups is not significantly different.

It can be concluded that the results of this research were similar to those of other published studies^16,34,43^ and consistent with the goals and hypotheses of our plan due to the higher levels of Th1 cytokine production, IFN-γ and IL-2, in comparison to Th2 cytokines, IL-10 and IL-4; considering our purpose in the selection of specific sequences of E6, E7, and L1 genes which stimulate cytotoxic T lymphocytes (CTLs); and considering the archaeosome properties for the activation of the cellular immune system.

Furthermore, the location of the antigen in liposome affects immunity. Both surface localized and encapsulated antigens induce T-cell response. The advantage of the first case is availability as well as fast and easier release from archaeosome, while the enclosed antigens need disruption within the vesicle in order to be available. The results demonstrated significant differences between the efficacies of archaeosome-containing DNA vaccine compared with when DNA was injected nakedly, similar to others studies. The cytokine profile represents switching the immune response toward Th1 using the archaeosome, well demonstrated in the reduction in the size of the tumor. Moreover, in this study, no significant difference between negative control groups is shown.

The impacts of each vaccine in the induction of immune responses has been studied by measuring the tumor size. There was a significant difference between the tumor volumes of test groups compared to control groups. Finally, with regard to the profile of cytokine production and their results in inhibiting tumor growth, the synergistic effect of the L1/E6/E7 recombinant gene and archaeosome can be concluded. The findings clearly indicated the positive effect of some of the structures on the reduction of the HPV-E6/E7-expressing tumors size. By monitoring the tumor size in the remaining mice, it was well-established that archaeosome-formulated pDNAs are well able to stop tumor growth in the animal. In general, the results of spleen cell cytokine secretion and measuring tumor volume in this study confirm that the candidate DNA vaccine in formulations with archaeosome nanoparticles in both surface and encapsulation formulation has created the best anti-tumor response in comparison with other vaccinated groups.

In order to further study the possible effect of L1/E6/E7-archaeosome vaccine on cervical cancer, we detected apoptosis signals in tumor sections with the TUNEL method. TUNEL staining data indicated that apoptosis occurred in tumor cells from mice immunized with L1/E6/E7 DNA vaccine. It is interesting that apoptosis in the tumor cells of immunized mice clearly increased after the administration of both plasmid DNA immunogens adsorbed onto or encapsulated inside archaeosome nanoparticles. However, no apoptosis occurred in mice immunized with the PBS control group. The result suggested that there was some relation between the combination treatment of L1/E6/E7 recombinant gene/archaeosome and apoptosis.

## Conclusion

Humans are constantly exposed to archaeal-like lipids, so archaeosome as a natural adjuvant can be safe and suitable for human body. One of the main obstacles to many new adjuvant technologies, especially those that are able to augment potent immune response, is their toxicity, particularly at high doses. Archaeosomes lack toxicity and can be activate the immune system.

Simultaneous utilization of HPV 16 genes, L1, E6, and E7 in combination therapy with archaeosomes can be an excellent strategy for stimulate immune responses against existing HPV-16-associated malignancies, holding great promise for the therapeutic vaccine development.

This therapeutic approach offers a novel prospect for the improvement and rational design of potent and safe vaccines capable of eliciting T cell immunity against cancers and intracellular infections. Although there is a long way to reach the final result in the use of archaeosome, it seems that the application of this technology has its undeniable benefits. Archaeosomes have been introduced as highly valuable carrier for vaccines which, besides appropriate presentation of antigen to immunocompetent cells and improving antigen stability, they have the ability to overcome biological obstacles, such as skin and mucosa. Moreover, they provide controlled and slow release of antigens. In addition to the ability to induce a potent immune response by adjuvant property, archaeosome-based vaccines have features that are essential for the improvement of modern vaccine formulations. It is predictable that this therapeutic and prophylactic strategy will be increasingly employ in the near future successfully and can lead to major advances in vaccine development.

## Materials and Methods

### Plasmid DNA experiments

The recombinant DNA vaccine candidate (L1/E6/E7) utilized in this study was generated in our previous study^44^. To generate the recombinant construct (L1/E6/E7) encoding CTL-epitopes restricted by both human and mouse, the cellular antigenic regions of the human papillomavirus type 16 genes, containing L1, E6 and E7 selected and linked by a spacer of glutamic acid, alanine, alanine, alanine, lysine. The list of the amino acid sequences of the peptides can be found as Supplementary Table S1. The final DNA sequences was designed and optimized using bioinformatics software (see Supplementary Table S2), then synthesized by Macrogen company (korea). Synthesized gene sequences encoding cellular antigenic epitopes of the L1, E6 and E7 create 397 bp. This construct was subcloned into pIRES2-EGFP vector, coding the green fluorescence protein, and named pIRES- L1/E6/E7. Then, the construct expression in HEK293 cell line was verified via western blot. The applied primary antibodies included mouse monoclonal Anti-HPV16 L1 antibody [CamVir 1] (ab69). Secondary antibodies were goat anti- Mouse IgG H&L (HRP) (Abcam). Binding signals were visualized with TMB substrate (Sigma-Aldrich, T0565).The result of western blot provided as a supplementary file (see Supplementary Fig. S1).

In current study, the pIRES-L1/E6/E7 and pIRES (mock plasmid) was used to construct archaeosome-plasmid DNA (pDNA) formulations. The competent cells of Escherichia coli DH5α strain were transformed via recombinant pDNA in the Luria-Bertani medium in the presence of kanamycin. Amplification and purification of plasmids were performed with a GeneAll® ExprepTM Plasmid SV mini (Korea). The quality and quantity of the pDNA were spectrophotometrically detected with a Thermo Scientific NanoDrop™ 2000/2000c, and electrophoresis was performed on a 1% agarose gel^45^. For the large-scale preparation of the plasmids, pIRES-L1/E6/E7 and pIRES were performed by ion exchange chromatography with a Plasmid Mega kit (QIAGEN, Cat No./ID: 12181) according to the manufacturer’s instructions.

### Expression of pIRES-L1/E6/E7-mediated GFP gene in the HEK293 cell

In current study, we examined the GFP protein expression using a fluorescence microscope. The recombinant DNA vaccine candidate (L1/E6/E7) utilized in this study was subcloned into pIRES2-EGFP vector. This vector contains the internal ribosome entry site (IRES; 1, 2) of the encephalomyocarditis virus (ECMV) between the Multiple Cloning Site (MCS) and the enhanced green fluorescent protein (EGFP) coding region. This permits both the gene of interest (cloned into the MCS) and the EGFP gene to be translated from a single bicistronic mRNA.

The HEK293 (Human Embryonic Kidney 293) cells were cultivated in DMEM (Gibco, USA) cell-culture medium without any antibiotics, supplemented with 10% fetal bovine serum (FBS, Gibco, USA), 1 mM sodium pyruvate, 2 mM glutamine, and 2 mM nonessential amino acids, and incubated at 37 °C with 5% CO2 and 95% humidity. The cells were passaged at 80% confluency and 5 × 10^4^ cells per well seeded into 24-well culture plates, and incubated under standard culture conditions for 24 hour. Two hours before transfection the culture medium was renewed. Sub-confluent HEK293 cells were transfected with pIRES-L1/E6/E7 using Lipofectamine 2000 (Invitrogen, USA) according to the manufacturer’s protocol and incubated at 37 °C with 5% CO2 for 24 h. Empty pDNA was used as the control in these experiments. The medium was replaced 6 h after the transfection to remove plasmids not internalized by cells. Twenty-four hours post-transfection, the transfected cells were analyzed by immunofluorescence microscopy (IF).

### Strain and archaebacterial growth conditions

*Halobacterium salinarum* (*H. salinarum*) was provided by Pasteur Institute (Tehran, Iran). The cells were grown aerobically at 40 °C in the modified growth medium for the cultures described by Choquet *et al*., containing (g/l): (NH4)_2_SO_4_ (3), K_2_HPO_4_ (0.5), MgSO_4_⋅7H_2_O (0.5), Ca(NO_3_)_2_⋅4H_2_O (0.01), and KCl (0.1). Sulfur (10 g/l) and 0.1% yeast extract were added to the basal medium and adjusted to pH 7.2 using sulfuric acid. Cultures were incubated in rotary shakers for 10 days at 40 °C, monitored by absorbance at 660 nm, harvested in the late stationary phase, and stored at −20 °C as cell pastes prior to lipid extraction.

### Extraction of lipids

Total polar lipids were prepared from frozen and thawed pastes of *H. salinarum* by the modified method of Bligh and Dyer and the total polar lipid fraction collected by precipitation by cold acetone. Cell paste was thawed by water and solvents were added to obtain a volume of methanol/chloroform/water at the ratio of 2:1:0.8 v/v and the mixture was stirred for 24 h at room temperature. The suspension was centrifuged at 5000 g for 20 min in glass centrifuge bottles. The supernatants containing the lipids were combined and transferred into a separatory funnel. Subsequently, chloroform (CHCl3) was added to produce a biphasic system. The lower organic phase (chloroform layer) included the lipids, and the top methanol-water layer generally contained the non-lipid components. The chloroform layer was withdrawn, the volume of CHCl_3_ was depleted to dryness by rotary evaporation at 60 °C, and the lipid films thus obtained were dried under high vacuum. The lipid residue was dissolved into CHCl_3_ adding the minimum volume of methanol required, and the polar lipids were precipitated by adding ice-cold acetone (Choquet *et al*., 1992). After storage at −20 °C for 24 h, the white precipitate of total polar lipids was collected by centrifugation and dissolved in CHCl_3_. Lipid extracts dissolved in CHCl_3_ were stable indefinitely but were placed at 4 °C to minimize solvent evaporation over long storage periods and the resultant precipitation of lipids from concentrated extracts. The chloroform phase was dried with lyophilization, and the TPLs were stored at 4 °C until required.

### Transmission electron microscopy (TEM) studies

The formation and morphological characteristics of uncomplexed archaeosomes were analyzed with TEM. Archaeosome samples were examined by a negative staining technique using adsorption on a carbon-film–covered copper grid, stained with 2% (w/v) uranyl acetate, and investigated with an electron microscope (ZIESS, EM900, PHILPS, Germany) at an accelerating voltage of 60 kV.

### Encapsulation and surface localization of plasmid DNA in archaeosomes

Archaeosomes were prepared aseptically by hydrating the lyophilized TPL from *H. salinarum*. For the preparation of encapsulated plasmid DNA**-**archaeosome formulation, dried total polar lipids were hydrated at 37 °C in phosphate-buffered saline (PBS) supplemented with either plasmid DNA. The solutions were vortexed and hydration was allowed to proceed at 37 °C with shaking for 24 h. Empty archaeosome was formed following the same procedure. For the latter, manufacturing of the surface localization of plasmid on lipid vesicles, plasmid DNA was surface-bound by incubating empty archaeosomes at 37 °C with shaking overnight. To improve complex formation between pDNA and archaeosomes, CaCl_2_ as a helper molecule was also added into the medium^20^. In the end, the suspensions were sonicated at room temperature for three times 5 min with intervals of 3 min using a sonication bath (Ultrasonic Cleaner Set WUC-D10H, Korea). Any plasmid not associated with the vesicles was removed by centrifugation (25000 × g for 1 h), followed by washing the vesicle pellet twice in PBS. The formulations were stored at 4 °C until use.

### Particle size and zeta potential measurements

The average size and zeta potential of the archaeosomal formulations were measured using a Zetasizer Nano ZS (Malvern Instruments, Malvern, UK). Sample size were detected by dynamic light backscattering. All measurements were conducted at 27 °C and in triplicate.

### *In vivo* tumor formation in C57BL/6 mice

Inbred female C57BL/6 mice five to six weeks of age were purchased from the Pasteur Institute (Tehran, Iran) and maintained in the animal facility of the Tarbiat Modares University (Tehran, Iran). All animal procedures were performed according to the approved protocols and in accordance with recommendations for the proper use and care of the Animal Ethics Committee of the Faculty of Medical Sciences, Tarbiat Modares University. The approval No. IR.TMU.REC.1394.193.

TC-1 cells were purchased from Pasteur Institute of Iran (Tehran, Iran). The cells were propagated in DMEM (Gibco, USA) cell-culture medium supplemented with 10% fetal bovine serum (FBS, Gibco, USA) and 100 IU/ml of penicillin, 100 µg/ml of streptomycin, 2 mM glutamine, 1 mM sodium pyruvate, and 2 mM nonessential amino acids, and incubated at 37 °C with 5% CO_2_ and 95% humidity. The cells were harvested at 80% confluency, and 7 × 10^5^ cell suspensions in 100 μl of PBS were inoculated subcutaneously in the right flank of C57BL/6 mice.

### Immunization protocol

Six animal groups, each consisting of eight pathogen-free female C57BL/6 mice, were handled according to animal care ethics committee of the Faculty of Medical Sciences, Tarbiat Modares University, (the approval no. IR.TMU.REC.1394.193) and immunized according to the schemes presented in Table 2. All vaccine formulations were administered subcutaneously (sc) in 0.1 ml volume in PBS. As the negative control, PBS was injected to control group. Two booster immunizations were performed one and two weeks after the first immunization. Tumor growth was monitored three times a week by inspection and palpation. Mice were euthanized one week after the last immunization (i.e., Day 28) and spleens were collected for the evaluation of Ag-specific immune responses.

**Table 2:** Experimental groups immunized with different vaccine formulations.

Groups	Compound (100 μl)	Abbreviate
1	pDNA-surface localized archaeosome	pDNA + (S)Arch
2	pDNA-encapsulated archaeosomes	pDNA + (E)Arch
3	pDNA alone (L1/E6/E7 recombinant gene)	pDNA
4	archaeosomes alone (antigen-free archaeosomes)	Arch
5	pIRES (Control group)	Mock Plasmid
6	PBS (Control group)	PBS

The vaccine dose per mice injected as follows:

Dose of pDNA alone (L1/E6/E7 recombinant gene): 50 µg; Dose of archaeosomes alone (antigen-free archaeosomes): 1 mg; Dose of Archeasome/DNA mixture: pDNA-surface localized archaeosome AND pDNA-encapsulated archaeosomes: 1 mg of Archeasome containing 50 µg of pDNA.

Days of immunization (Days after TC1 inoculation): 7, 14, and 21.

Route of administration: Subcutaneous.

### Cell cytotoxicity assay

In order to assess cell cytotoxicity, lactate dehydrogenase (LDH) level was determined in splenocytes. LDH is an intracellular enzyme found in the cells of many body tissues. one week after the last immunization, a single-cell suspension of splenocytes was prepared and used as effector cells. A precise number of TC-1 cells (4 × 10^4^) as target cells were co-cultured with effector cells in a volume of 100 μl in phenol red-free RPMI containing 3% FBS at effector/target ratios of 25:1. Generation of effector CD8 + T cells (CTLs) was assayed by the LDH release assay in triplicate in 96-well plates according to the manufacturer’s instructions (LDH Cytotoxicity Detection Kit Plus, Roche, Germany). Optical density was read at the wavelength of 492 nm (A492), and cytotoxicity was calculated according to the formula:

$$ \% \,{\rm{Cytotoxicity}}=({\rm{Experimental}}\,{\rm{value}}-{\rm{Low}}\,{\rm{control}}/{\rm{High}}\,{\rm{control}}-{\rm{Low}}\,{\rm{control}})\times 100.$$

The median value was used for the calculations of the cytotoxicity value. All determinations were performed in triplicate.

### Antigen-specific proliferation of splenocytes and cytokine assays

To assay proliferation, single-cell suspensions of splenocytes from vaccinated and control mice were obtained one week after the last treatment by gentle homogenization. Selective lysis of erythrocytes was performed with Tris-buffered ammonium chloride (20 mM Tris, 160 mM NH4Cl, pH 7.2), (Sigma Chemical Co.) for 5 min at room temperature, and the pelted splenocytes were resuspended in RPMI supplemented with 20% FBS, 2mM L-glutamine, 100 U/ml of penicillin and 100 µg/ml of streptomycin. The cells at the concentration of 2 × 10^6^ cells/ml were seeded in triplicate in 24-well plates; stimulated with 10 μg/ml of concanavalin A (Con A; C7275 Sigma, USA) or 10 µg/ml of Escherichia coli LPS (L2630 Sigma, USA); and then incubated at 37 °C with 5% CO_2_. Culture supernatants were collected after 72 h for the measurement of IFN-γ, IL-2, IL-4, and IL-10 cytokine secretion using commercial DuoSet enzyme-linked immunosorbent (ELISA) cytokine assay kits (R&D system, Minneapolis, MN) according to the manufacturer’s instructions. The lower limit for the detection of the cytokine was 32 pg/ml. Values were presented as pg cytokine/ml (mean ± SD, n = 5).

### *In situ* apoptotic cell detection

Tumor cell apoptosis was determined *in vivo* with the *In Situ* Cell Death Detection kit (Roche Molecular Biochemicals, Mannheim, Germany) according to the manufacturer’s instructions. Briefly, seven days following the final administration, the mice were euthanized and histological studies were conducted. Autopsied tumors from animals were fixed in 10% phosphate-buffered formalin. The formalin-fixed paraffin-embedded tumor tissue was sectioned in three different regions from the central part of the tumor, and deparaffinized. Each section was rehydrated through a graded series of ethanol and double distilled water, washed in PBS, incubated with PBS containing 20 μg/ml of proteinase K for 20 min, and permeabilized with 0.1% Triton x-100 and 0.1% sodium citrate. The sections were then incubated in TUNEL solution at 37 °C for 1 h. Finally, the samples were washed with PBS and observed under a fluorescent microscope (×40). Apoptotic cells were counted on a basement membrane of seminiferous tubules in each group.

### Statistical analysis

Data were expressed as mean ± SD. One-way ANOVA and two-way ANOVA with Tukey’s test were performed for data comparisons. The GrapPad Prism software version 7.04 was used to plot charts. Asterisks indicated groups are significantly different from each other (*P ≤ 0.05; **P ≤ 0.01; ***P ≤ 0.001; ****P ≤ 0.0001).

### Ethics approval and consent to participate

The animal experiments followed the guidelines of the Laboratory Animal Ethical Commission of the Faculty of Medical Sciences, Tarbiat Modares University. The approval No. IR.TMU.REC.1394.193.

## Supplementary information

Supplementary Figure S1.

Supplementary Table S1.

Supplementary Table S2.

## Data Availability

All relevant data are included within the paper and supporting information files. Please contact the corresponding author for material availability.

## References

[1] Kaarthigeyan K (2012). Cervical cancer in India and HPV vaccination. Indian journal of medical and paediatric oncology: official journal of Indian Society of Medical & Paediatric Oncology.

[2] Stanley MA (2012). Genital human papillomavirus infections: current and prospective therapies. Journal of General Virology.

[3] Stern PL (2012). Therapy of human papillomavirus-related disease. Vaccine.

[4] Ferlay, J. *et al*. Global cancer observatory: cancer today. *Lyon, France: International Agency for Research on Cancer* (2018).

[5] Bharti AC, Singh T, Bhat A, Pande D, Jadli M (2018). Therapeutic strategies for human papillomavirus infection and associated cancers. Front Biosci (Elite Ed).

[6] Hancock G, Hellner K, Dorrell L (2018). Therapeutic HPV vaccines. Best Practice & Research Clinical Obstetrics & Gynaecology.

[7] Lowy DR (2016). HPV vaccination to prevent cervical cancer and other HPV-associated disease: from basic science to effective interventions. The Journal of clinical investigation.

[8] Jia Y, Krishnan L, Omri A (2015). Nasal and pulmonary vaccine delivery using particulate carriers. Expert opinion on drug delivery.

[9] Stanley MA (2012). Epithelial cell responses to infection with human papillomavirus. Clinical microbiology reviews.

[10] Levine RM, Pearce TR, Adil M, Kokkoli E (2013). Preparation and characterization of liposome-encapsulated plasmid DNA for gene delivery. Langmuir.

[11] Krishnan L, Dicaire CJ, Patel GB, Sprott GD (2000). Archaeosome vaccine adjuvants induce strong humoral, cell-mediated, and memory responses: comparison to conventional liposomes and alum. Infection and immunity.

[12] Brown DA, Venegas B, Cooke PH, English V, Chong PL-G (2009). Bipolar tetraether archaeosomes exhibit unusual stability against autoclaving as studied by dynamic light scattering and electron microscopy. Chemistry and physics of lipids.

[13] Kaur G, Garg T, Rath G, Goyal AK (2016). Archaeosomes: an excellent carrier for drug and cell delivery. Drug delivery.

[14] Benvegnu T, Lemiègre L, Cammas-Marion S (2009). New generation of liposomes called archaeosomes based on natural or synthetic archaeal lipids as innovative formulations for drug delivery. Recent patents on drug delivery & formulation.

[15] Patel, G. B. & Chen, W. In *Nanocarrier technologies* 17–40 (Springer, 2006).

[16] Schwendener RA (2014). Liposomes as vaccine delivery systems: a review of the recent advances. Therapeutic advances in vaccines.

[17] Vazzana, M., Fangueiro, J. F., Faggio, C., Santini, A. & Souto, E. B. Archaeosomes for Skin Injuries. *Carrier-Mediated Dermal Delivery: Applications in the Prevention and Treatment of Skin Disorders, Pan Stanford Publishing, Singapore*, 323–355 (2017).

[18] Krishnamachari Y, Geary SM, Lemke CD, Salem AK (2011). Nanoparticle delivery systems in cancer vaccines. Pharmaceutical research.

[19] Faisal SM (2011). Immunostimulatory and antigen delivery properties of liposomes made up of total polar lipids from non-pathogenic bacteria leads to efficient induction of both innate and adaptive immune responses. Vaccine.

[20] Attar A, Ogan A, Yucel S, Turan K (2016). The potential of archaeosomes as carriers of pDNA into mammalian cells. *Artificial cells*. nanomedicine, and biotechnology.

[21] Zapatka, M. *et al*. The landscape of viral associations in human cancers. *bioRxiv*, 465757 (2018). 10.1038/s41588-019-0558-9 PMC8076016 32025001

[22] Araldi RP (2018). The human papillomavirus (HPV)-related cancer biology: An overview. Biomedicine & Pharmacotherapy.

[23] Bolhassani A (2018). Therapeutic HPV Vaccines: Immunogenicity, Efficacy and Safety. *HPV Infections: Diagnosis*. Prevention, and Treatment.

[24] Schiller JT, Castellsagué X, Garland SM (2012). A review of clinical trials of human papillomavirus prophylactic vaccines. Vaccine.

[25] Lin Y-L, Borenstein LA, Selvakumar R, Ahmed R, Wettstein FO (1992). Effective vaccination against papilloma development by immunization with L1 or L2 structural protein of cottontail rabbit papillomavirus. Virology.

[26] Saade F, Petrovsky N (2012). Technologies for enhanced efficacy of DNA vaccines. Expert review of vaccines.

[27] Amador-Molina A, Hernández-Valencia J, Lamoyi E, Contreras-Paredes A, Lizano M (2013). Role of innate immunity against human papillomavirus (HPV) infections and effect of adjuvants in promoting specific immune response. Viruses.

[28] Bellone S (2009). Human papillomavirus type 16 (HPV-16) virus-like particle L1-specific CD8+ cytotoxic T lymphocytes (CTLs) are equally effective as E7-specific CD8+ CTLs in killing autologous HPV-16-positive tumor cells in cervical cancer patients: implications for L1 dendritic cell-based therapeutic vaccines. Journal of virology.

[29] Liu WJ (2002). Codon modified human papillomavirus type 16 E7 DNA vaccine enhances cytotoxic T-lymphocyte induction and anti-tumour activity. Virology.

[30] Lin C-T (2006). A DNA vaccine encoding a codon-optimized human papillomavirus type 16 E6 gene enhances CTL response and anti-tumor activity. Journal of biomedical science.

[31] Mirshahabi H, Soleimanjahi H, Pourpak Z, Meshkat Z, Hassan ZM (2012). Production of human papilloma virus type 16 e6 oncoprotein as a recombinant protein in eukaryotic cells. Iranian journal of cancer prevention.

[32] Moghimipour, E., Kargar, M., Ramezani, Z. & Handali, S. The potent *in vitro* skin permeation of archaeosome made from lipids extracted of Sulfolobus acidocaldarius. *Archaea* **2013** (2013). 10.1155/2013/782012 PMC3888715 24453698

[33] Singh, M. *Vaccine adjuvants and delivery systems*. (John Wiley & Sons, 2007).

[34] Krishnan, L. & Sprott, G. D. Archaesome vaccine adjuvants for cross-priming CD8+ T cell immunity. *Vaccine adjuvants and delivery systems. Edited by Singh M. Hoboken, NJ: John Wiley & Sons*, 263–294 (2007).

[35] Réthoré, G. *et al*. Archaeosomes based on synthetic tetraether-like lipids as novel versatile gene delivery systems. *Chemical Communications*, 2054-2056 (2007). 10.1039/b618568a 17713076

[36] Garnett, M. C. Gene-delivery systems using cationic polymers. *Critical Reviews™ in Therapeutic Drug Carrier Systems* **16** (1999). 10706442

[37] Liu Z, Winters M, Holodniy M, Dai H (2007). siRNA delivery into human T cells and primary cells with carbon‐nanotube transporters. Angewandte Chemie.

[38] Allen TM, Cheng K, Wilson W, Hare JI, Laginha KM (2006). Pharmacokinetics and pharmacodynamics of lipidic nano-particles in cancer. Anti-Cancer Agents in Medicinal Chemistry (Formerly Current Medicinal Chemistry-Anti-Cancer Agents).

[39] Senior J, Gregoriadis G (1982). Stability of small unilamellar liposomes in serum and clearance from the circulation: the effect of the phospholipid and cholesterol components. Life sciences.

[40] Inoh Y, Nagai M, Matsushita K, Nakanishi M, Furuno T (2017). Gene transfection efficiency into dendritic cells is influenced by the size of cationic liposomes/DNA complexes. European Journal of Pharmaceutical Sciences.

[41] Simões S (2000). Human serum albumin enhances DNA transfection by lipoplexes and confers resistance to inhibition by serum. Biochimica et Biophysica Acta (BBA)-Biomembranes.

[42] Krishnan L, Sprott GD (2008). Archaeosome adjuvants: immunological capabilities and mechanism (s) of action. Vaccine.

[43] Haq K, Jia Y, Krishnan L (2016). Archaeal lipid vaccine adjuvants for induction of cell-mediated immunity. Expert review of vaccines.

[44] Saadat, P. *et al*. Co-Administration of Anti-Angiogenic Peptide and DNA Vaccine in Cervical Cancer Tumor Model. *International Journal of Cancer Management* **10** (2017).

[45] Karimi H, Soleimanjahi H, Abdoli A, Shirmohammadi M (2017). Application of Archaeosome Nanoparticles as a DNA Vaccine Delivery System and Evaluation of its Effect in a C57BL/6 Tumor Model. Pathobiology Research.

