# Supplementary material for: Combination therapy using human papillomavirus L1/E6/E7 genes and archaeosome: a nanovaccine confer immuneadjuvanting effects to fight cervical cancer
Source: Sci Rep. 2020 Apr 1;10:5787. doi: 10.1038/s41598-020-62448-3 (PMC7113280; doi:10.1038/s41598-020-62448-3)
Supplement: Supplementary file 1 — Supplementary Figure S1. [file 41598_2020_62448_MOESM1_ESM.pdf]

Supplementary Figure S1

Title: Combination therapy using human papillomavirus L1/E6/E7 genes and archaeosome: a nanovaccine confer immuneadjuvanting effects to fight cervical cancer

Hesam Karimi [1], Hoorieh Soleimanjahi [1*], Asghar Abdoli [2], Razieh Sadat Banijamali [1]

1. Department of Virology, Faculty of Medical Sciences, Tarbiat Modares University, Tehran, Iran
2. Department of Hepatitis and AIDS, Pasteur Institute of Iran, Tehran, Iran

* Corresponding Author: Hoorieh Soleimanjahi, PhD; Professor – Tel: [+98] 21 82883561- Email: soleim_h@modares.ac.ir

Western blot analysis

The recombinant pIRES-L1/E6/E7 DNA vaccine utilized in this study encodes a protein with molecular weight of ~14.3 kDa includes 128 amino acid. To confirm the recombinant DNA vaccine candidate (pIRES-L1/E6/E7), SDS-PAGE electrophoresis and western blot were applied to verify the expression of recombinant gene proteins. Sub-confluent HEK293 cells were transfected with pIRES-L1/E6/E7 using lipofectamine 2000 (Invitrogen, USA) according to the manufacturer's protocol and incubated at 37 °C with 5% CO_2 for 24 h. The medium was replaced 6 h after the transfection to remove plasmids not internalized by cells. Twenty-four hours post-transfection, the cells were analyzed by immunofluorescence microscopy (IF). Then the cells were harvested and analyzed with western blot assay. The applied primary antibodies included mouse monoclonal Anti-HPV16 L1 antibody [CamVir 1] (ab69). Secondary antibodies were goat anti- Mouse IgG H&L (HRP) (Abcam). Binding signals were visualized with TMB substrate (Sigma-Aldrich, T0565).

Supplementary figure S1.

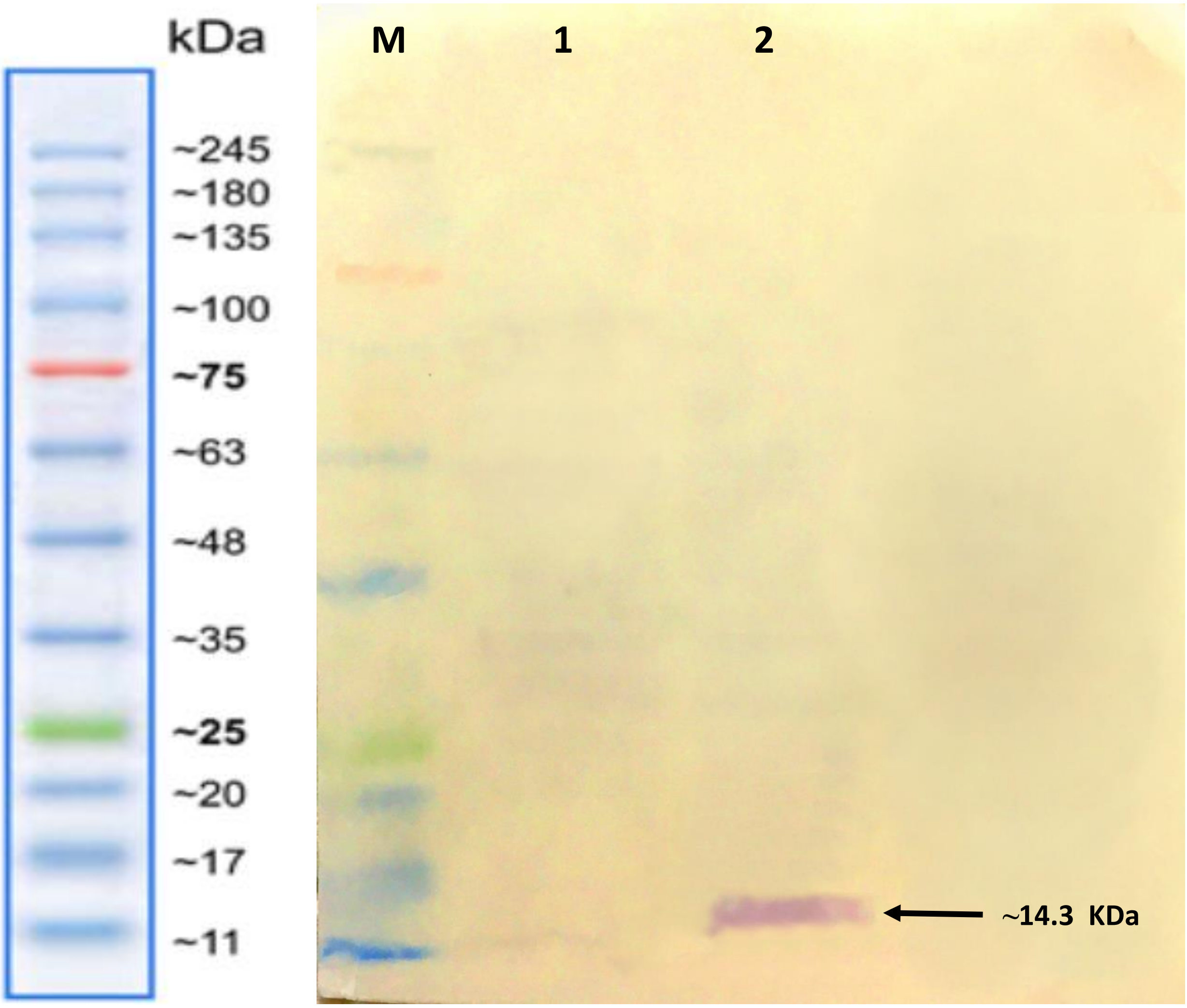

Fig. S1. The recombinant DNA vaccine (pIRES-L1/E6/E7) expression in HEK293 cell line evaluated by western blotting with mouse monoclonal Anti-HPV16 L1 antibodies. Lane M: protein marker, lane 1: negative control, lane 2: The recombinant pIRES-L1/E6/E7 DNA vaccine encodes a protein with molecular weight of ~14.3 kDa includes 128 amino acid.
