# Supplementary material for: Combination therapy using human papillomavirus L1/E6/E7 genes and archaeosome: a nanovaccine confer immuneadjuvanting effects to fight cervical cancer
Source: Sci Rep. 2020 Apr 1;10:5787. doi: 10.1038/s41598-020-62448-3 (PMC7113280; doi:10.1038/s41598-020-62448-3)
Supplement: Supplementary file 2 — Supplementary Table S1. [file 41598_2020_62448_MOESM2_ESM.pdf]

Supplementary Table S1

Title: Combination therapy using human papillomavirus L1/E6/E7 genes and archaeosome: a nanovaccine confer immuneadjuvanting effects to fight cervical cancer

Hesam Karimi [1], Hoorieh Soleimanjahi [1*], Asghar Abdoli [2], Razieh Sadat Banijamali [1]

1. Department of Virology, Faculty of Medical Sciences, Tarbiat Modares University, Tehran, Iran
2. Department of Hepatitis and AIDS, Pasteur Institute of Iran, Tehran, Iran

* Corresponding Author: Hoorieh Soleimanjahi, PhD; Professor – Tel: [+98] 21 82883561- Email: soleim_h@modares.ac.ir

Table S1. The amino acid sequences of the antigenic epitopes of the HPV 16 L1, E6, and E7 genes

Type	Protein	Sequence of antigenic epitopes
HPV16	L1	AGVDNRECISMDYKQTQLCLIGCKPPI
HPV16	E6	SEYRHYCYSLYGTTLEQQYNKPLCDLL
HPV16	E7	RAHYNIVTFRAHYNIVTFRAHYNIVTF

To generate the recombinant construct (L1/E6/E7) encoding CTL-epitopes restricted by both human and mouse, the cellular antigenic regions of the human papillomavirus type 16 genes, containing L1, E6 and E7 selected and linked by a spacer of glutamic acid, alanine, alanine, alanine, and lysine. The final synthesized gene sequences (397 bp) encodes a protein with molecular weight of ~14.3 kDa includes 128 amino acid.
