# Supplementary material for: Combination therapy using human papillomavirus L1/E6/E7 genes and archaeosome: a nanovaccine confer immuneadjuvanting effects to fight cervical cancer
Source: Sci Rep. 2020 Apr 1;10:5787. doi: 10.1038/s41598-020-62448-3 (PMC7113280; doi:10.1038/s41598-020-62448-3)
Supplement: Supplementary file 3 — Supplementary Table S2. [file 41598_2020_62448_MOESM3_ESM.pdf]

Supplementary Table S2

Title: Combination therapy using human papillomavirus L1/E6/E7 genes and archaeosome: a nanovaccine confer immuneadjuvanting effects to fight cervical cancer

Hesam Karimi [1], Hoorieh Soleimanjahi [1*], Asghar Abdoli [2], Razieh Sadat Banijamali [1]

1. Department of Virology, Faculty of Medical Sciences, Tarbiat Modares University, Tehran, Iran

2. Department of Hepatitis and AIDS, Pasteur Institute of Iran, Tehran, Iran

* Corresponding Author: Hoorieh Soleimanjahi, PhD; Professor – Tel: [+98] 21 82883561- Email: soleim_h@modares.ac.ir

To generate the recombinant construct (L1/E6/E7) encoding CTL-epitopes restricted by both human and mouse, the cellular antigenic regions of the human papillomavirus type 16 genes, containing L1, E6 and E7 selected and linked by a spacer of glutamic acid, alanine, alanine, alanine, lysine.

To confirmation that the CTL epitopes are restricted to both human and mouse, the CTL epitopes were screened using IEDB and VaxiJen servers for predicting MHC Class-I and II alleles. IEDB was first used to predict the highest scoring epitopes of chimer which binds with maximum number of MHC Class-I alleles. The antigenic score of each epitope was evaluated using VaxiJen 2 server. The antigenicity score for total epitopes with percentile rank below 1 was analyzed using VaxiJen Server.

Table S2: Mouse & Human MHC-I binding prediction using IEDB & Vaxijen servers

Start position	Peptide epitope	length	Allele	Percentile rank	Antigenicity score	Antigenicity
Mouse MHC-I						
96	RAHYNIVTF	9	H-2-Db	0.2	0.5919	+
105	RAHYNIVTF	9	H-2-Db	0.2	0.5919	+
114	RAHYNIVTF	9	H-2-Db	0.2	0.5919	+
102	VTFRAHYNI	9	H-2-Kb	0.76	0.9571	+
111	VTFRAHYNI	9	H-2-Kb	0.76	0.9571	+
Human MHC-I						
48	KSEYRHYCY	9	HLA-A*01:01	0.31	1.8399	+
61	TTLEQQYNK	9	HLA-A*11:01	0.22	0.4815	+
55	CYSLYGTTL	9	HLA-A*24:02	0.58	0.4301	-
91	EAAAKRAHY	9	HLA-A*25:01	0.88	0.9203	+
96	RAHYNIVTF	9	HLA-B*35:01	0.6	0.5919	+
105	RAHYNIVTF	9	HLA-B*35:01	0.6	0.5919	+
114	RAHYNIVTF	9	HLA-B*35:01	0.6	0.5919	+
